# Optic tract injury after closed head traumatic brain injury in mice: A model of indirect traumatic optic neuropathy

**DOI:** 10.1371/journal.pone.0197346

**Published:** 2018-05-10

**Authors:** Nathan K. Evanson, Fernanda Guilhaume-Correa, James P. Herman, Michael D. Goodman

**Affiliations:** 1 Division of Pediatric Rehabilitation Medicine, Cincinnati Children’s Hospital Medical Center, Cincinnati, Ohio, United States of America; 2 Department of Pediatrics, University of Cincinnati, Cincinnati, Ohio, United States of America; 3 Department of Psychiatry and Behavioral Neuroscience, University of Cincinnati, Cincinnati, Ohio, United States of America; 4 Department of Surgery, University of Cincinnati, Cincinnati, Ohio, United States of America; Uniformed Services University, UNITED STATES

## Abstract

Adult male C57BL/6J mice have previously been reported to have motor and memory deficits after experimental closed head traumatic brain injury (TBI), without associated gross pathologic damage or neuroimaging changes detectable by magnetic resonance imaging or diffusion tensor imaging protocols. The presence of neurologic deficits, however, suggests neural damage or dysfunction in these animals. Accordingly, we undertook a histologic analysis of mice after TBI. Gross pathology and histologic analysis using Nissl stain and NeuN immunohistochemistry demonstrated no obvious tissue damage or neuron loss. However, Luxol Fast Blue stain revealed myelin injury in the optic tract, while Fluoro Jade B and silver degeneration staining revealed evidence of axonal neurodegeneration in the optic tract as well as the lateral geniculate nucleus of the thalamus and superior colliculus (detectable at 7 days, but not 24 hours, after injury). Fluoro Jade B staining was not detectable in other white matter tracts, brain regions or in cell somata. In addition, there was increased GFAP staining in these optic tract, lateral geniculate, and superior colliculus 7 days post-injury, and morphologic changes in optic tract microglia that were detectable 24 hours after injury but were more prominent 7 days post-injury. Interestingly, there were no findings of degeneration or gliosis in the suprachiasmatic nucleus, which is also heavily innervated by the optic tract. Using micro-computed tomography imaging, we also found that the optic canal appears to decrease in diameter with a dorsal-ventral load on the skull, which suggests that the optic canal may be the site of injury. These results suggest that there is axonal degeneration in the optic tract and a subset of directly innervated areas, with associated neuroinflammation and astrocytosis, which develop within 7 days of injury, and also suggest that this weight drop injury may be a model for studying indirect traumatic optic neuropathy.

## Introduction

Traumatic brain injury (TBI) is one of the leading causes of death and disability, and leads to annual costs of at least $60 billion in the United States alone [1]. Because of the magnitude of TBI-related morbidity and mortality, dedicated research has pursued an understanding of the pathophysiology of TBI, across the spectrum of mild to more severe injuries. Animal models have been valuable in gaining understanding of pathologic changes after brain injury. Investigators have used multiple approaches to modeling TBI in rodents, including open skull methods such as controlled cortical impact and fluid percussion models, and closed skull models such as blast TBI and weight drop closed head models, among others [2].

One TBI model that has been developed is a closed head weight-drop model [3, 4]. This model was adapted to be performed in mice, with scalp intact [5]. It was noted that mice undergoing TBI in this modified weight drop model developed blood-brain barrier permeability in the acute phase after injury, and serum and cytokine profiles consistent with inflammation. On the rotarod test, mice undergoing TBI were able to remain on a rotating rod for less time than sham-treated animals, a result which is consistent with motor impairments. Similarly, in the novel object recognition test, post-TBI mice were less able to recognize the novel object, as shown by a significantly lower ratio of time with the novel object to time with the familiar object than was observed in sham animals [5]. These deficits appeared to be due to inflammation secondary to the injury [6]. However, in contrast to the findings by Chen et al., there was no gross pathological evidence of injury or volume loss, and neuroimaging (by magnetic resonance and diffusion tensor imaging) likewise did not show evidence of injury in the milder injury group. Thus, specific brain areas affected by the injury were not identifiable by our previously utilized methods [5].

The current studies were undertaken to gain a greater understanding of tissue level damage that may underlie the functional deficits seen in this model of TBI. We hypothesized that, since neurologic performance deficits are evident in these mice after TBI, there must be brain damage present that could potentially be detectable using more sensitive histological techniques. Also, since previous work demonstrated evidence of pro-inflammatory cytokine production [6], there should be histologic evidence of neuroinflammation in these animals. Thus, we undertook the use of histologic measures to find evidence of more subtle brain injury after mild closed head TBI.

## Materials and methods

### Animals

**Fig 1:**
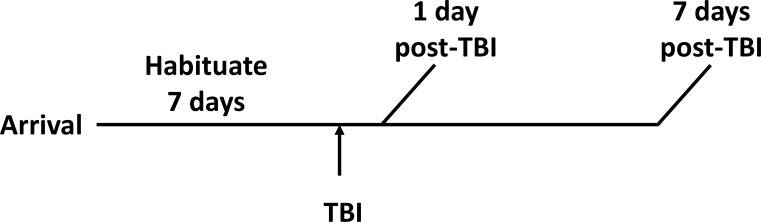
Experimental timeline. Animals were divided into two main cohorts, with study performed at 1 day post-TBI or 7 days post-TBI. Animals were sacrificed at 1 day or 7 days post-TBI (n = 8 sham and 8 TBI at each time point). TBI: traumatic brain injury.

These studies were performed in adult 8 week old male C57BL/6J mice (Jackson Labs, Bar Harbor, ME). All procedures were approved by the University of Cincinnati Institutional Animal Care and Use Committee. Animals were kept in pressurized individually ventilated cages, 4 animals per cage, with ad libitum access to water and standard rodent chow. Cages were kept in temperature- and humidity-controlled holding rooms, with alternating 12 hour light:dark schedules. Procedures were performed during the subjective light phase. For histologic measures, 8 mice per group were used (n = 32). For weight gain measures, 16 mice per group were used (n = 64 total). Fig 1 shows a schematic of the experimental procedures.

### Traumatic brain injury

Experimental TBI was induced using a closed head weight drop method. This approach is similar to other closed head approaches [3, 4], but with the scalp left intact, as previously described [5]. Briefly, animals were anesthetized using 2% isoflurane, weighed, then positioned in the TBI apparatus. Injury was induced by dropping a 400g rod 1.5 cm onto the animal’s head, with impact centered approximately over bregma. Immediately following impact, animals were observed until righting reflexes returned (up to 10 minutes, with mean approximately 5 minutes), then returned to their home cages. Sham treated animals were anesthetized and weighed, then monitored until righting reflexes returned (within 10 seconds after weighing), then returned to home cages.

### Histology

For immunohistochemical analysis, animals were sacrificed by pentobarbital overdose, then perfused transcardially with saline followed by 4% paraformaldehyde. Brains were removed and immersion post-fixed in paraformaldehyde for about 7 days, then saturated with sucrose by immersion in a 30% sucrose solution. Brains were then frozen with dry ice and cut into 30 micron sections using a sliding microtome (Leica, Buffalo Grove, IL), then stored at -20°C in cryoprotectant solution (30% sucrose, 1% polyvinylpyrrolidone, and 30% ethylene glycol, in 50mM sodium phosphate buffer, pH 7.4) until used for staining.

Histologic staining was performed using cresyl violet Nissl stain. Neuronal degeneration stains used were Fluoro Jade B (Histo-Chem, Jackson, AR) according to the manufacturer’s directions [7] and silver neurodegeneration stain using the NeuroSilver II kit (FD Neurotechnologies, Columbia, MD), according to the manufacturer’s instructions. Myelin was visualized using Luxol Fast Blue staining kit (Abcam, Cambridge, MA), according to the manufacturer’s instructions. Immunohistochemistry was performed using rabbit primary antibodies raised against glial fibrillary acidic protein (GFAP, DAKO, Santa Clara, CA, catalog # Z0334) or ionized calcium-binding adaptor molecule (Iba-1, Synaptic Systems, Goettingen, Germany, catalog # 234 003); or mouse monoclonal antibody raised against neuronal nuclei antigen (NeuN, EMD Millipore, Burlington, MA, catalog # MAB377). Anti-NeuN was used at 1:500 dilution and anti GFAP and Iba-1 were both used at 1:2000 dilution. Fluorescent secondary antibody was Cy-3 conjugated goat anti-mouse (Jackson Immunochemicals, West Grove, PA, catalog # 115-166-072) for NeuN or Cy-3 conjugated donkey anti-rabbit for other primaries (Jackson, catalog # 711-165-152), both used at 1:500 dilution. After staining, slides were cover-slipped using antifading polyvinyl alcohol mounting medium (Sigma-Aldrich, St. Louis, MO).

### Microscopy and image analysis

Slides were photographed using an Axio Imager.Z1 microscope with Apotome attachment (Leica Microsystems, Buffalo Grove, IL). All slides within a particular comparison group were photographed by a blinded observer on the same day, using the same exposure time and magnification. GFAP images, obtained with immunofluorescence, were analyzed semi-quantitatively using ImageJ software [8], by measuring mean gray level in rectangular samples within the regions of interest. Four non-overlapping samples were obtained within each region of interest, and the mean value was taken from each animal. Immunofluorescence-labeled Iba-1 stained slides were photographed and thresholded using NIS Elements software (Nikon, Melville, NY). The thresholded areas of cell somata, in μm^2^, were automatically measured in each region of interest, using the NIS Elements software, and mean thresholded soma area was calculated and used as an estimation of microglial soma size. All image analysis was performed by a blinded observer. Illustrative photomicrographs found in the figures in this manuscript were adjusted for contrast and brightness, but otherwise were unaltered. Photomicrographs used for image analysis were not altered in any way prior to image analysis.

### Micro-CT

To qualitatively evaluate how the mouse skull deforms under dorsal-ventral compression, a mouse was euthanized using carbon dioxide inhalation, then analyzed by micro-CT on an ImTek micro CT system (ImTek, Knoxville, TN), at the Cincinnati Children’s Hospital Medical Center Imaging Research Center. Image analysis was performed using the Amira software package (FEI, Hillsboro, OR). Post-mortem scans were done at rest, then repeated using a wooden clamp to cause dorsal-ventral skull compression, in approximately the same plane as impact from the TBI device.

### Statistics

Statistical analysis was done using the SigmaPlot software package (Systat, San Jose, CA). Results are reported as mean +/- SEM. Weight change was analyzed by 2-way analysis of variance (ANOVA) with repeated measures, and post-hoc analysis was done using the Holm-Sidak method. Histologic measures were analyzed by unpaired t-test, comparing TBI to sham within each time point. Data were transformed if needed, so as to not violate equal variance or normality assumptions. Significance level was set *a priori* at p < 0.05.

## Results

### Survival and weight loss

**Fig 2:**
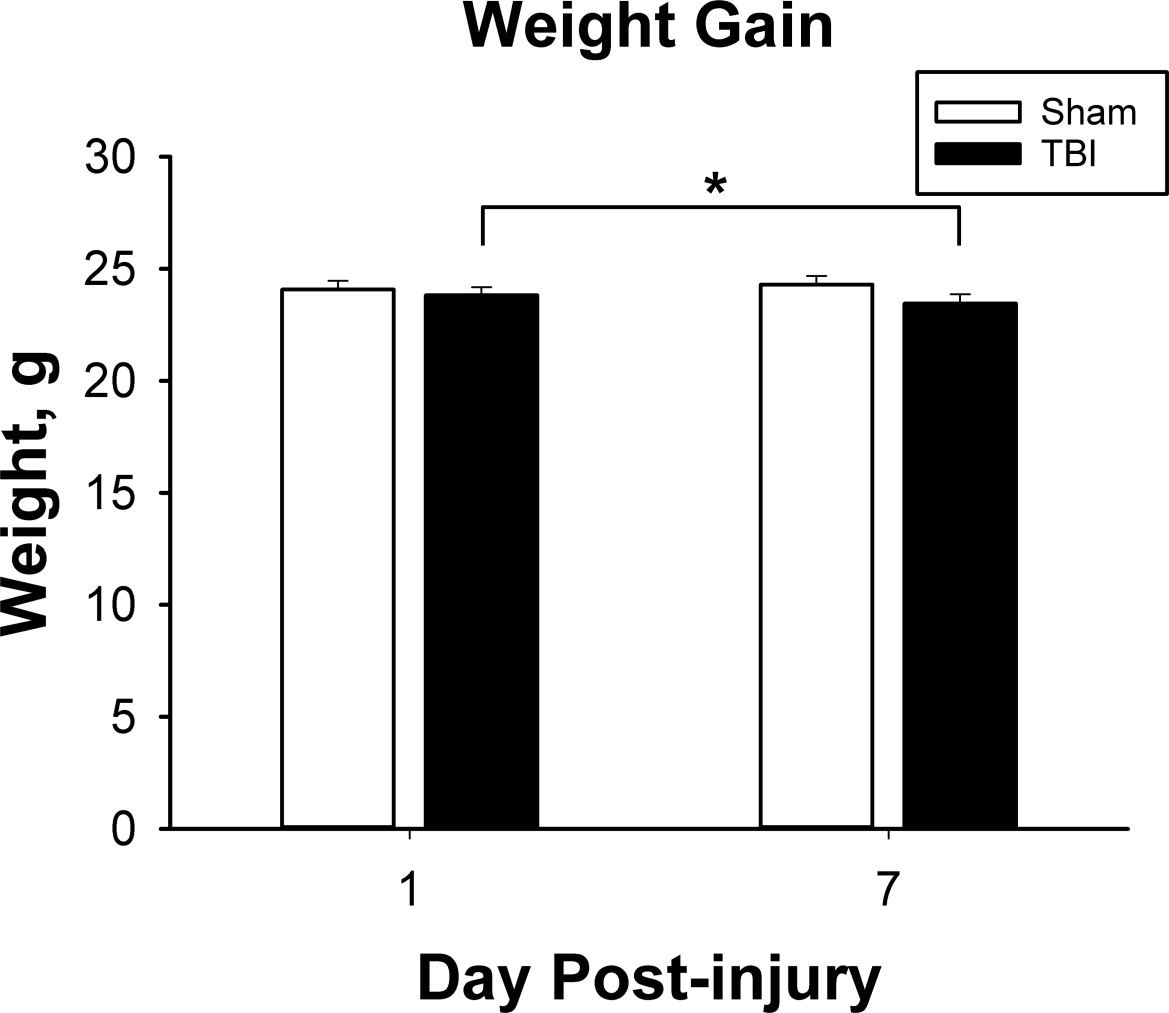
Weight change. Animals with TBI lost a slight amount of weight between pre-surgery weights and 7 days post-injury. There was no significant difference overall between TBI and sham animals, and there was no difference between day 1 and day 7 sham animals (n = 16 per group at each time point). * p < 0.05 vs pre-surgery weight.

Eight week old mice were subjected to TBI by weight drop. Overall mortality rate immediately after injury was approximately 10%. Animals that died did not regain normal respiratory function after impact, and expired within the first minute after TBI. Out of the animals that regained righting reflex, survival rate was 100% to the end of the experimental time period. Within the first approximately 24 hours, injured mice displayed obviously decreased activity, which recovered to baseline after the first 24 hours. After this there were no obvious signs of injury by gross observation. Over the first week of the experiment, weight was stable in the sham animals, and decreased a small but statistically significant amount in the TBI animals. (Treatment x day interaction, F_1,30_ = 12.83, p < 0.001; p = 0.004 for the difference between TBI day 1 and day 7). There was not a significant difference between TBI and sham animals overall (F_1,30_ = 1.05, p = 0.31 for TBI effect) or at either time point. (Fig 2)

### Histologic measures of damage

**Fig 3:**
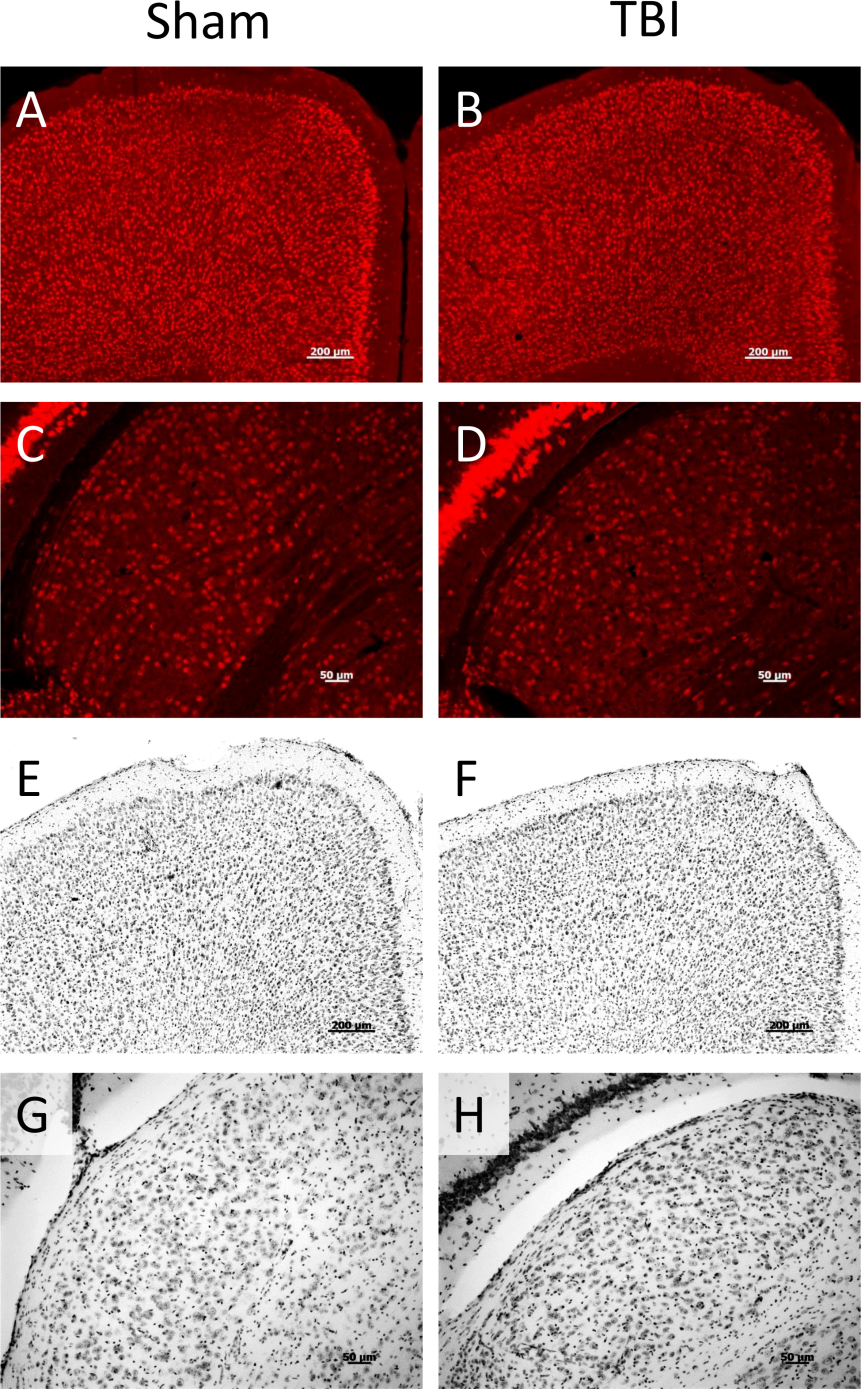
NeuN and Nissl staining. There was no discernible difference in the appearance of NeuN or Nissl staining between TBI and sham animals 7 days post-injury. A, C, E, G are from sham animals and B, D, F, H are from TBI animals. Typical NeuN patterns are shown for prefrontal cortex (A, B) and lateral geniculate nucleus of the thalamus (LGN, C, D). Typical appearance of Nissl stained sections are also illustrated, from prefrontal cortex (E, F) and LGN (G, H).

**Fig 4:**
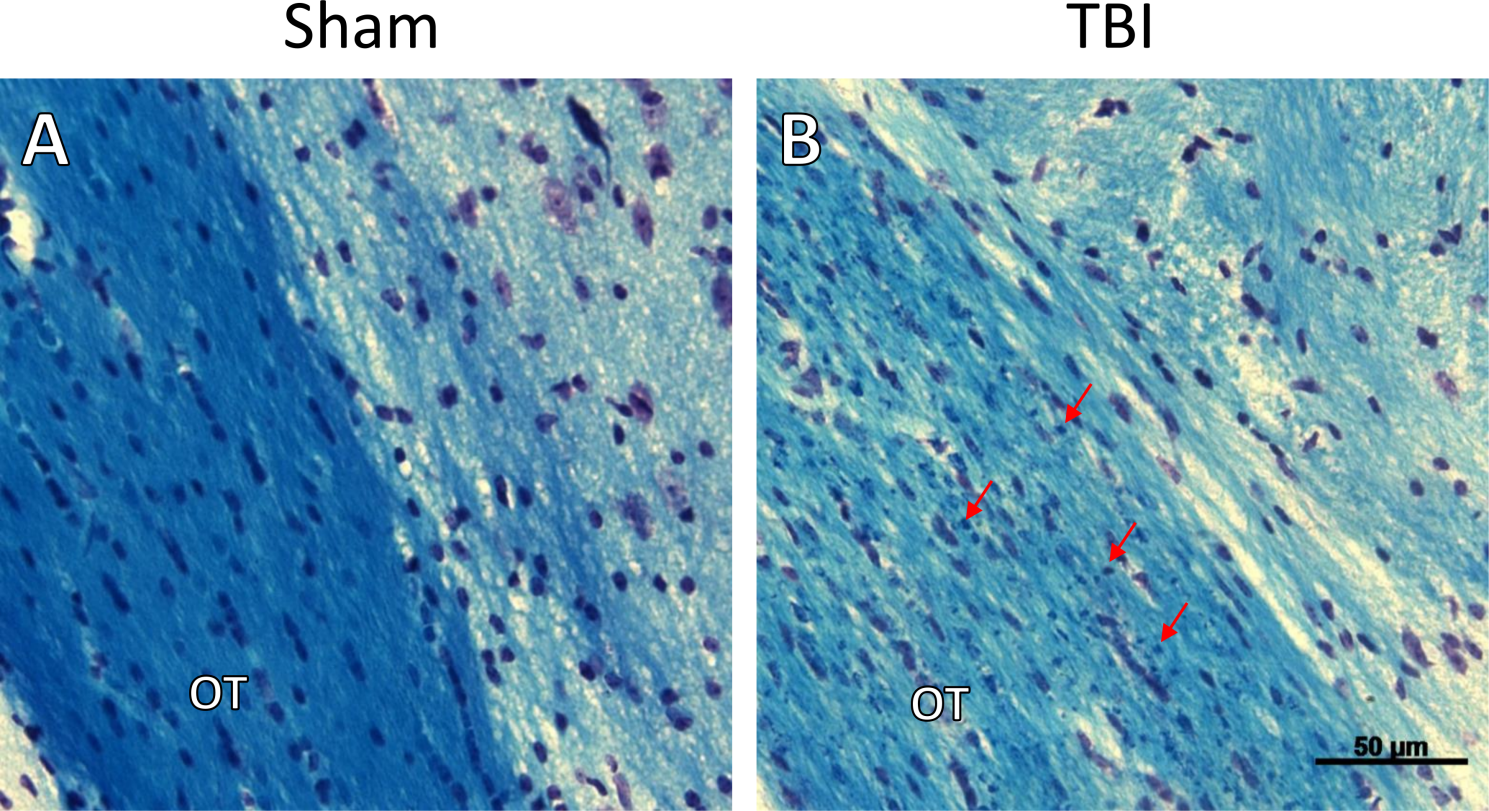
Luxol Fast Blue myelin stain. Representative micrographs from sham (A) or TBI (B) animals are shown, centered over the optic tract. There is a subjective decrease in the intensity of staining in the optic tract of TBI animals, 7 days after injury. In addition, there are globular collections of positive staining seen within the optic tract, consistent with the appearance of myelin after white matter injury. For clarity, several of these myelin globules are indicated by arrows in part B of the figure. OT–optic tract. Scale bar indicates 50 μm.

**Fig 5:**
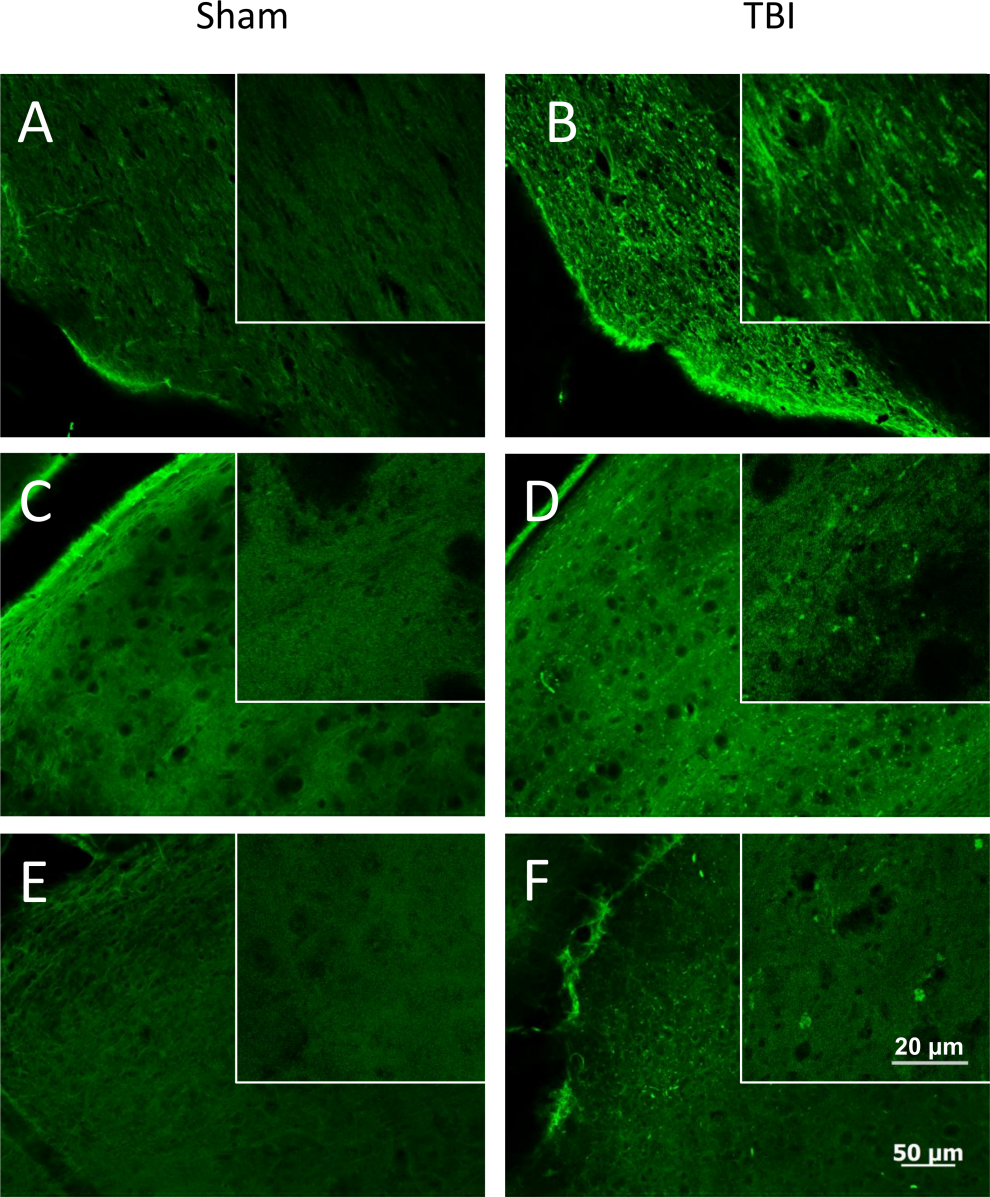
Fluoro Jade B staining for neurodegeneration. Representative staining from sham (A, C, E) and TBI (B, D, F) animals, from the 7 day post-injury group. Sections from optic tract (A, B), LGN (C, D), and superior colliculus (E, F) are shown. Inset images are higher magnification photographs from the same region. There is a punctate staining pattern present in all three areas in animals with TBI, 7 days after injury, which is absent in TBI mice 1 day post-injury and in sham animals at either time point. The 50 μm scale bar applies to all low-magnification micrographs and the 20 μm bar applies to all inset micrographs.

**Fig 6:**
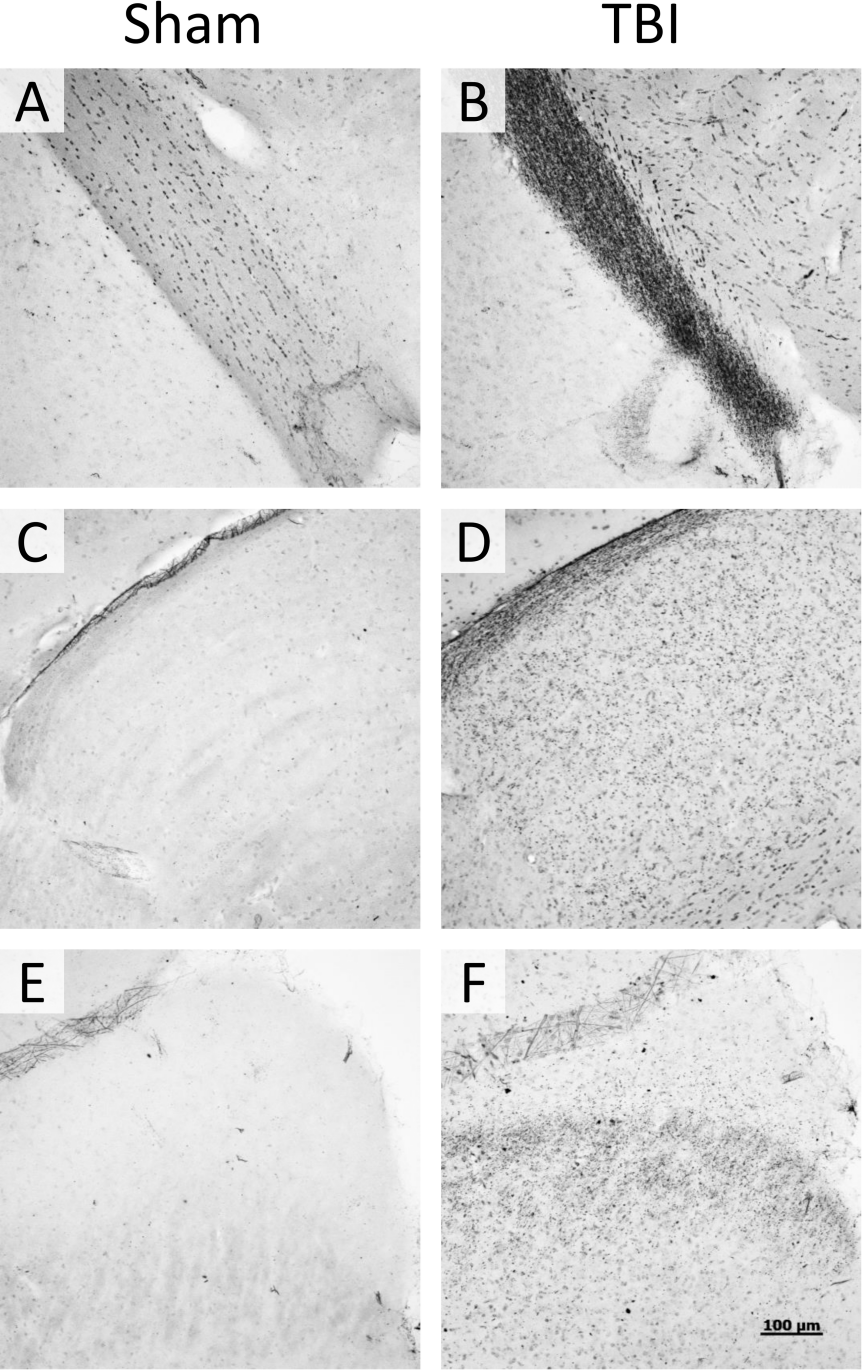
NeuroSilver degeneration stain. Representative staining from sham (A, C, E) and TBI (B, D, F) animals, using a silver stain for neurodegeneration (NeuroSilver II stain). Sections from optic tract (A, B), LGN (C, D), and superior colliculus (E, F) are shown. There is positive staining in a similar pattern to that seen in Fig 5 for Fluoro Jade B staining.

Consistent with previously reported results [5], we saw no evidence of gross anatomic damage or changes in Nissl stain or NeuN staining (Fig 3). Using other histologic measures, however, we found clear evidence of myelin injury and axonal degradation. Luxol Fast Blue myelin stain revealed overall lighter staining in the optic tract, as well as globular myelin staining (Fig 4). This appearance is consistent with that previously described in white matter injury, using this stain [9]. Fluoro Jade B staining revealed bilateral damage that was most obvious in the optic tract, and visible 7 days post-injury but not 24 hours after injury. In addition to the optic tract, there was also positive staining present in the lateral geniculate nucleus of the thalamus (LGN) and the superior colliculus (SC). The staining pattern in the LGN and SC was punctate and diffuse, but did not fill somata (see Fig 5). Apart from optic tract, LGN, and SC in the TBI group 7 days post-injury, there was no significant difference in staining between TBI and sham animals in other regions examined (white matter: lateral olfactory tract, anterior commissure, corpus callosum, fornix, alveus, cingulum, habenular commissure, posterior commissure, or cerebral peduncle; gray matter: infralimbic/prelimbic cortex, hippocampus, suprachiasmatic nucleus, visual cortex, or temporal association area). Using NeuroSilver stain for neurodegeneration, results were similar to those seen using Fluoro Jade B, with positive staining found in the optic tract, LGN, and SC (Fig 6).

**Fig 7:**
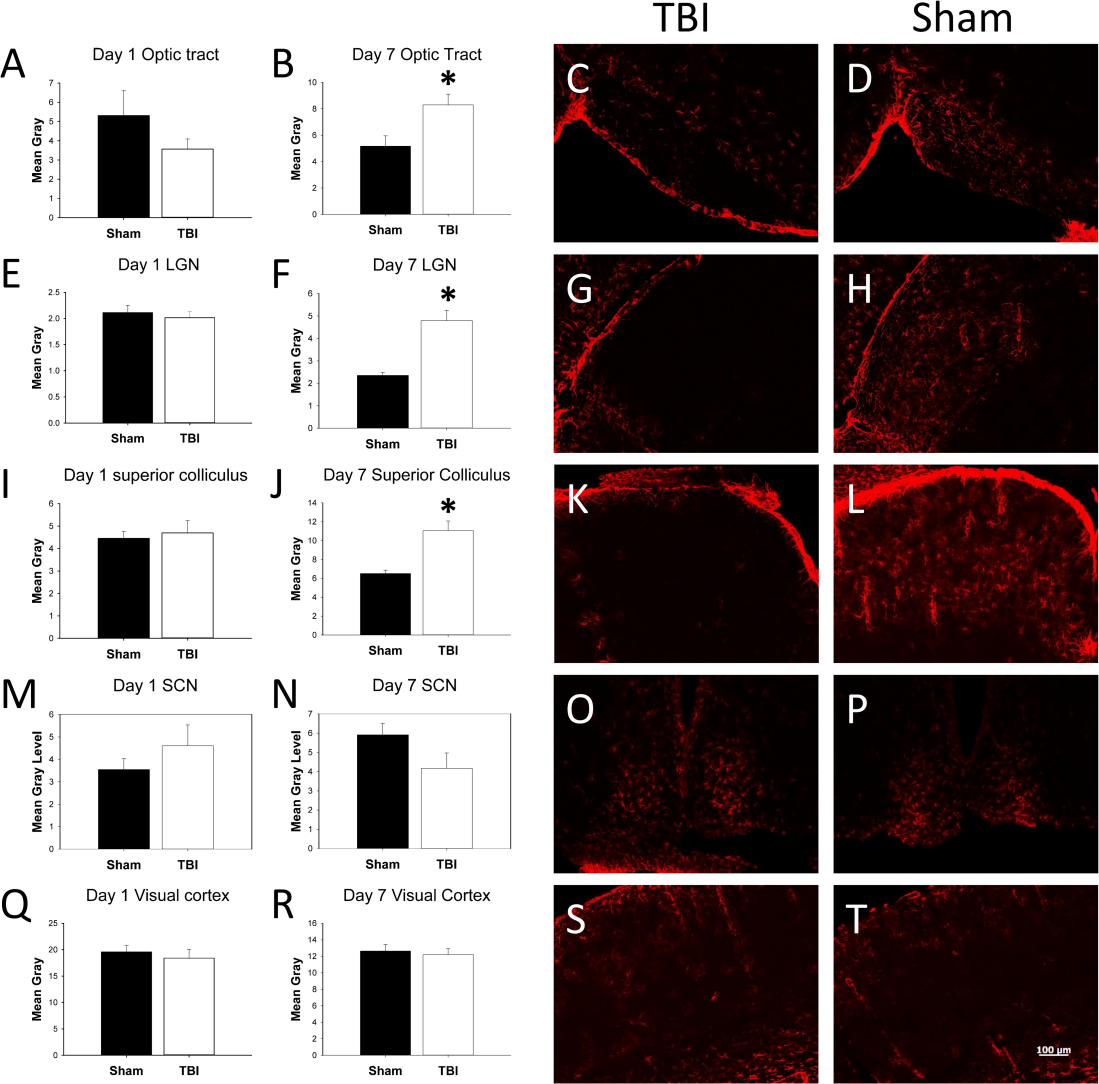
GFAP staining. Mean gray levels from TBI and sham animals in optic tract, LGN, SC, SCN, or visual cortex at either 1 day (A, E, I, M, Q) or 7 days (B, F, J, N, R) post-injury (n = 8 mice per group at each time point). * p < 0.05 vs sham group in the same day. Also shown are representative photomicrographs from each of these areas in sham (C, G, K, O, S) and TBI (D, H, L, P, T) animals, at 7 days post-injury.

Given evidence for neurodegeneration, we then tested for astrogliosis, using immunofluorescence analysis of glial fibrillary acidic protein (GFAP), which was analyzed in anterior commissure, corpus callosum, lateral olfactory tract, optic tract, LGN, SC, suprachiasmatic nucleus (SCN), visual cortex, somatosensory cortex, temporal association area, and infralimbic cortex. We found evidence for increased GFAP immunoreactivity in the same areas that stained positive for Fluoro Jade B, again at 7 days post-injury but not 1 day post-injury (Fig 7). Using semi-quantitative measurements across a number of regions, we found that 24 hours after TBI, there was no significant change in GFAP expression in TBI vs sham animals (results found in Table 1). However, at 7 days post-injury, we found increased GFAP expression specifically in optic tract (t(14) = -2.76, p = 0.02), LGN (t(13) = -5.57, p < 0.001), and SC (t(14) = -4.19, p < 0.001) (Table 2).

**Table 1:** GFAP day 1 mean gray measurements.

Location	Sham	(SEM)	TBI	(SEM)
Anterior Commissure	10.60	(0.93)	9.54	(1.16)
Corpus callosum	11.56	(1.07)	11.67	(1.32)
LOT	19.03	(2.12)	21.55	(3.43)
Optic tract	5.31	(1.29)	3.57	(0.52)
LGN	2.11	(0.14)	2.02	(0.12)
Superior colliculus	4.46	(0.31)	4.70	(0.55)
SCN	3.55	(0.49)	4.61	(0.93)
Visual cortex	19.56	(1.21)	18.40	(1.67)
SSC	28.70	(1.94)	26.50	(1.31)
TAA	19.14	(1.05)	17.67	(1.09)
Infralimbic Cortex	10.76	(0.91)	9.87	(0.52)

There were no significant differences in mean gray measurement from GFAP immunohistochemistry in the day 1 group. Quantities reported are in arbitrary units. LOT: lateral olfactory tract; LGN: lateral geniculate nucleus of the thalamus; SCN: suprachiasmatic nucleus SSC: somatosensory cortex; TAA: Temporal association area. These results are based on n = 8 animals per group.

**Table 2:** GFAP day 7 mean gray measurements.

Location	Sham	(SEM)	TBI	(SEM)
Anterior Commissure	7.07	(0.70)	6.47	(0.68)
Corpus callosum	13.55	(0.93)	14.00	(1.82)
LOT	12.07	(1.26)	11.70	(1.18)
Optic tract^a^	5.17	(0.78)	8.30	(0.82)
LGN^a^	2.35	(0.13)	4.79	(0.46)
Superior colliculus^a^	6.52	(0.34)	11.07	(1.03)
SCN	5.91	(0.60)	4.16	(0.81)
Visual cortex	12.65	(0.79)	12.20	(0.72)
SSC	17.85	(1.61)	17.10	(0.82)
TAA	16.10	(0.76)	15.10	(1.17)
Infralimbic Cortex	7.87	(0.61)	7.05	(0.45)

There was a significant increase in GFAP expression in Optic tract, LGN, SC. Quantities reported are in arbitrary units. LOT: lateral olfactory tract; LGN: lateral geniculate nucleus of the thalamus; SCN: suprachiasmatic nucleus; SSC: somatosensory cortex; TAA: Temporal association area. These results are based on n = 8 animals per group.

^a^ p < 0.05 vs sham animals.

**Fig 8:**
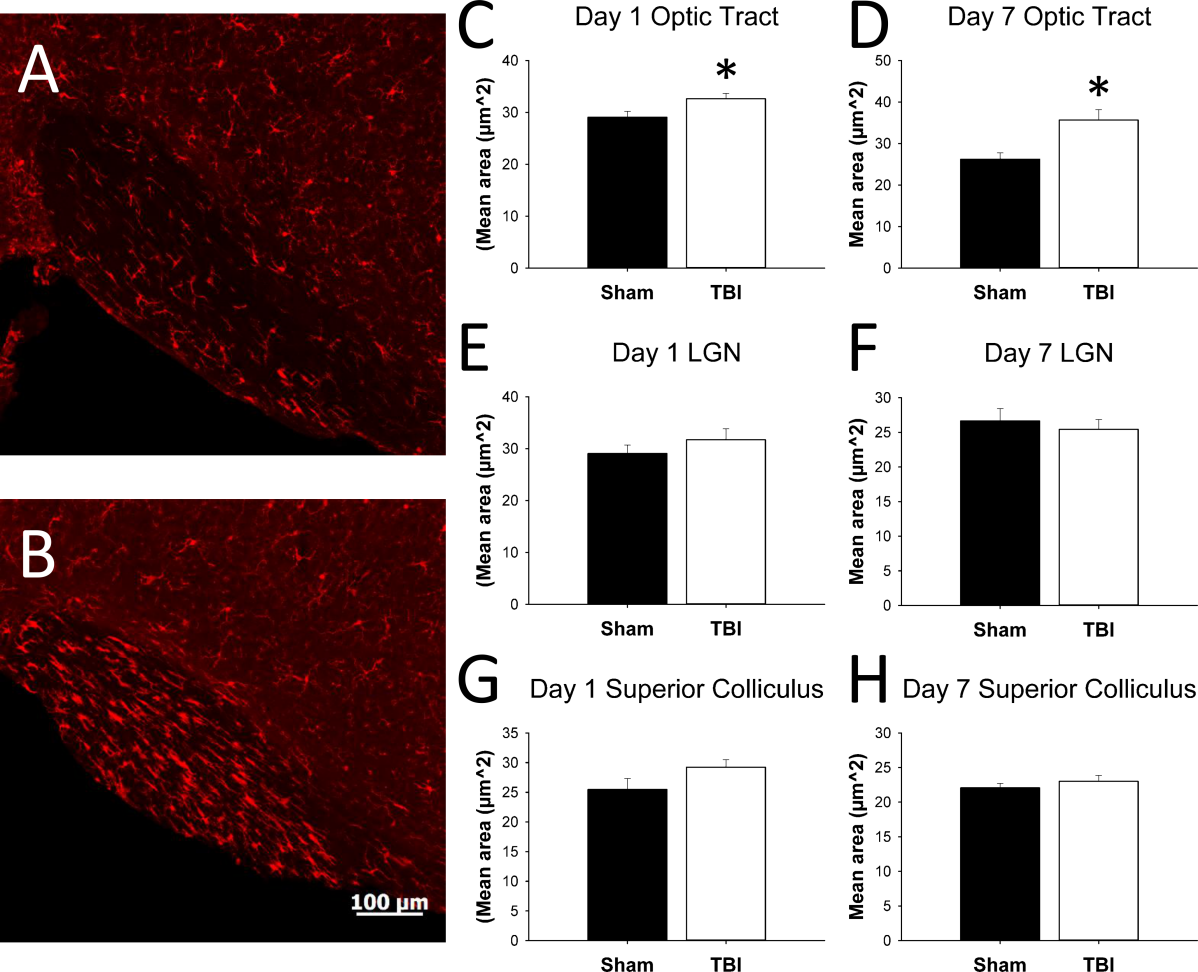
Iba-1 staining and microglial morphology analysis. Representative photomicrographs of the optic tract of sham (A) and TBI (B) animals, 7 days post-injury. (C–H) show mean area of microglia in optic tract, LGN, and SC (n = 8 per group at each time point). * p < 0.05.

Degeneration and astrogliosis suggest the potential for local inflammation. We used Iba-1 staining to determine whether there were morphologic changes in microglia consistent with activation (Fig 8). Increases in microglia size were observed in the optic tract by 24 hours after injury (t(14) = -2.294, p = 0.04), as well as at 7 days after injury (t(14) = -3.25, p = 0.006). There were no significant changes in microglia area in SC or LGN at either time point.

### MicroCT study

**Fig 9:**
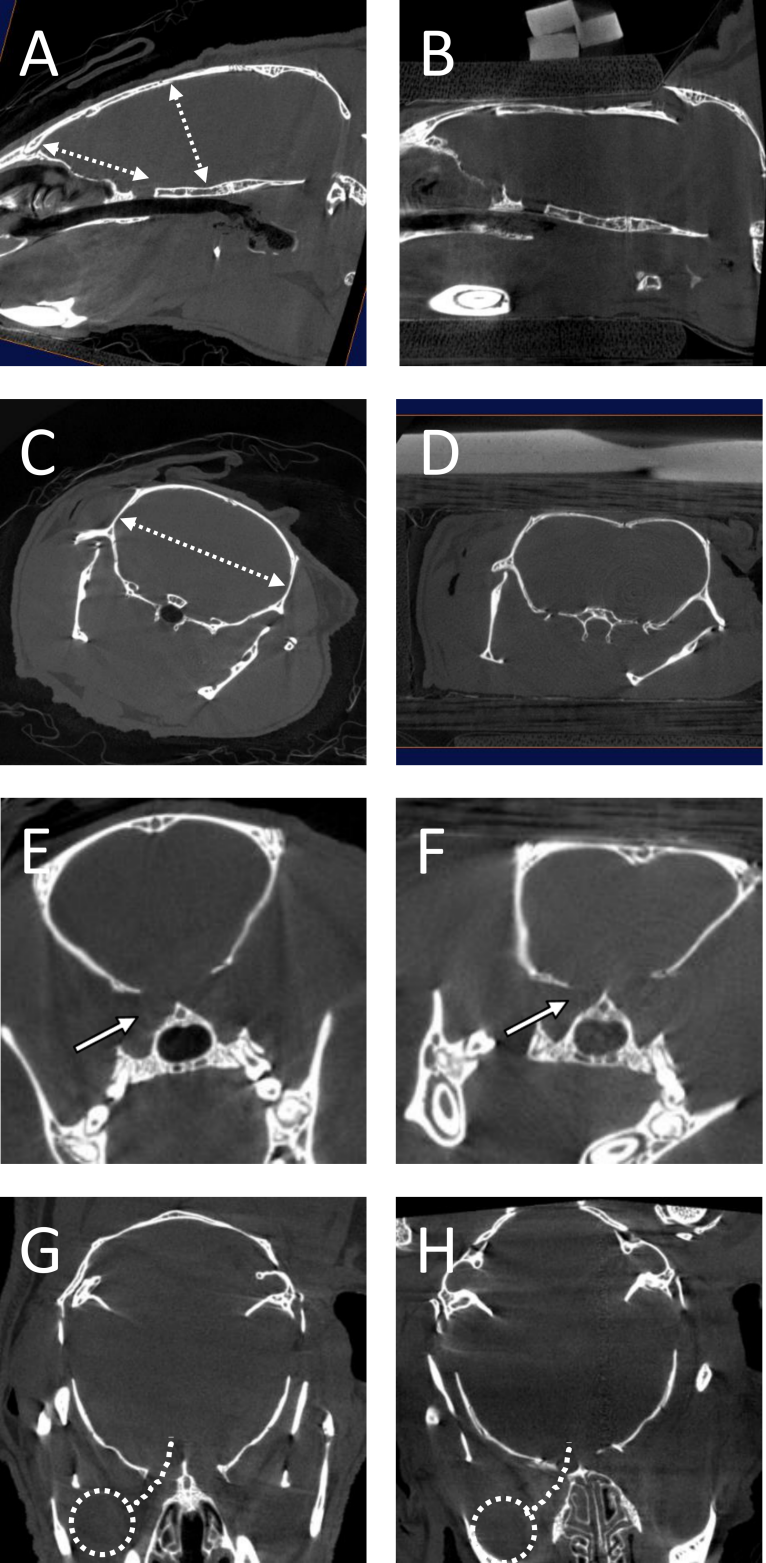
MicroCT analysis. Skull shape and measurements were evaluated using micro CT scanning either at rest (A, C, E, G), or under dorsal-ventral compression using a wooden clamp (B, D, F, H). Images were in the sagittal (A, B), coronal (C–F), or axial (G, H) planes. Measurements recorded in Table 3 were taken from these planes as indicated in A, C, and E. The approximate position of the eye and optic nerve are schematized in G and H.

Finally, to qualitatively understand how an impact on the skull might preferentially damage the optic tracts, a post-mortem mouse was examined using microCT, at rest and with the bilateral skull compressed through the dorsal-ventral axis using a wooden clamp, so as to decrease the dorsal-ventral skull measurement by about 1 mm. The distance from anterior cranium to the optic chiasm was measured (using the sella turcica as a bony landmark). This distance did not change with skull compression. There was a slight increase in the greatest medial-lateral measurement at the level of bregma. There was also about a 30% decrease in the diameter of the optic canal under compression. MicroCT results are shown in Fig 9 and Table 3.

**Table 3:** Micro CT measurements.

Measurement	Uncompressed	Compressed
Dorsoventral	5.25	4.4
Mediolateral	9.14	9.99
Apex to posterior sella	5.85	5.81
Coronal view optic canal	1.03	0.74

Measurements were done using Amira software, and are in mm. The planes in which these measurements were taken are illustrated in Fig 9. There was a decrease in dorsoventral measurement, consistent with the direction of skull compression. In addition, there was an increase in mediolateral width of the skull cavity, and a decrease in the width of the optic canal.

## Discussion

We have demonstrated in a murine closed head TBI model that there is reproducible damage not readily detectable by standard histological approaches, specific to the optic tract and some, but not all, brain regions receiving input from axons traveling in the optic tract. Damage is likely due to mechanical perturbation of the optic nerve entry into the cranium, and presents as frank axonal degeneration accompanied by astrocytosis and microgliosis.

We used histologic techniques including Nissl stain, Luxol Fast Blue, neurodegeneration silver stain, and Fluoro Jade B staining, as well as immunohistochemistry against NeuN, GFAP and Iba-1 to investigate the tissue-level response to closed head TBI in adult mice. NeuN is a DNA-binding protein that is found in the nuclei of most neuronal populations in the brain, and is thus used as an immunohistochemical marker for neurons [10]. Nissl stain likewise stains nuclei and somata of neurons, although it does not stain neuronal processes well. Both Nissl stain and NeuN stain can detect loss of neuronal cell bodies, based on morphologic changes or decreased staining [11]. Neither of these approaches stains cell processes [12], and both suffer from the limitation of looking for a loss of staining rather than positive staining, which can lead to missing a subtly positive result [13]. Luxol Fast Blue is a histologic stain for myelin, and reveals globular changes in the myelin after white matter injury [9]. Fluoro Jade B is a chemical derived from fluorescein, and specifically stains degenerating neurons and axons [7]. GFAP is the primary intermediate filament protein in astrocytes, and its expression is increased in cells involved in reactive astrocytosis [14]. Iba-1 is an ionized calcium binding protein expressed in microglia, and plays a role in regulating their function [15]. Overall, we did not observe differences either by gross brain pathology, or using NeuN or Nissl staining. Differences were evident, however, using Luxol Fast Blue, Fluoro Jade B, NeuroSilver, GFAP, and Iba-1 staining. These results suggest that there is not a gross loss of neurons or histologic architecture after this injury within the brain regions examined. There is, however, evidence of optic tract myelin injury, neuroinflammation, gliosis, and axonal degeneration. These findings are consistent with the clinical finding that deficits are seen after even mild TBI with normal neuroimaging (using computed tomography, magnetic resonance imaging, and/or diffusion tensor imaging), and agree with the assumption that there must therefore be damage present that is more subtle than that indicated by gross structural changes [16].

The pattern of histologic damage seen in this study is consistent with injury directly to fibers traveling in the optic tract, likely representing axons emanating from retinal ganglion cells. Damage is fairly extensive, including the development of degenerative changes in target regions for retinal ganglion cell projections, but not apparently including neuronal cell bodies in these regions. In addition, no Fluoro Jade B staining is observed in other white matter tracts, suggesting that axonal degeneration seen by Fluoro Jade B is likely limited to those traveling the optic tract, and does not represent a generalized white matter injury. Histologic consequences of this injury include axonal degeneration (by Fluoro Jade B staining) and increased gliosis (by increased GFAP staining) in the optic tract, as well as in the SC and LGN. Both of these regions receive direct innervation from the optic nerve/optic tract [17]. Interestingly, gliosis was not seen in Layer 4 of the visual cortex, which receives direct visual system input from the thalamus [18], but only indirect innervation from the optic nerve, via relay neurons in the LGN [17]. The absence of positive Fluoro Jade B staining in the visual cortex and in cell somata in the LGN and superior colliculus is consistent with absence of trans-synaptic degeneration in optic tract-innervated areas. This finding is also consistent with the absence of changes seen with NeuN and Nissl staining, which both support the conclusion that there is not significant loss of neurons in the brain regions examined. However, since both Fluoro Jade B and NeuroSilver stains detect degenerating axons [19], it is likely that the injury seen in brain tissues results from degenerating axons projecting from the optic tract. This conclusion is further supported by the observation that there is myelin injury in the optic tract as revealed by Luxol Fast Blue staining. It may be that more distant time points after injury would reveal degeneration in other vision-associated regions. In models of retinal or optic nerve damage such as elevated intraocular pressure [20] or optic nerve transection [21], LGN damage was noted by 1 week post-injury but became more pronounced at 4 weeks from injury or longer. Further studies will be needed to clarify the time course of changes in optic nerve target areas in this TBI model.

It is also interesting to note that not all targets of the optic nerve appear to display evidence of degenerative changes. The SCN, for example, is also heavily innervated by the optic nerve [22, 23], yet showed no evidence of neurodegeneration or of gliosis in the current studies. This finding raises the intriguing possibility that there is a differential susceptibility of axons in the optic tract to injury. The optic nerve is made up of axons from retinal ganglion cells, and over 30 different functional subtypes of these cells have been described [24]. One subset of retinal ganglion cells, the melanopsin-containing intrinsically photosensitive retinal ganglion cells, makes up most of the projections to the SCN in mice [25]. In light of the current results, it is interesting to note that intrinsically photosensitive retinal ganglion cells are more resistant to injury (albeit without enhanced axon regeneration) than other types of retinal ganglion cell in multiple animal models of optic nerve or retinal injury [26]. The mechanism of this enhanced survival is not known, but may be related to decreased susceptibility to glutamate excitotoxicity [26]. Because we found evidence of degenerating axons and gliosis in LGN and SC but not in SCN, it is possible that the optic tract injury found in our model spares some types of retinal ganglion cells (such as the melanopsin-containing variety, for example). Thus, this model may be a useful way to investigate factors that influence ganglion cell death or survival after traumatic optic nerve injury.

Traumatic axonal injury/diffuse axonal injury is a common component of clinical traumatic brain injuries, and is correlated with severity measures such as length of coma [27]. White matter injury severity is also predictive of cognitive deficits [28, 29] and chronic degenerative changes after TBI [30]. Modeling studies in primates have suggested that diffuse axonal injury is caused largely by shear/strain forces [31]. However, the mechanism of targeted axonal injury in the current weight drop TBI model is not clear. Thus, it was important to understand the physical mechanism that leads to this axonal injury. Micro CT imaging showed that there was not a significant increase of length through the anterior-posterior plane along the course of the optic nerve, and only a mild medial-lateral stretch of the skull cavity, which makes stretch injury less likely. In contrast, there was an approximately 30% decrease in the diameter of the optic canal under dorsal-ventral strain of the skull. The micro CT results suggested that the most likely mechanism for injury in this model is a crush injury of the optic nerve as it traverses the optic canal. The idea that this model presents a direct injury to the optic nerve is supported by the finding that histologic measures of damage were only seen in the LGN and superior colliculus, both of which are directly innervated by the optic nerve/optic tract [17]. However, we cannot fully rule out an element of stretch injury. Future studies will also be needed to confirm that the injury is sustained within the optic nerve proper, rather than more distally, in the optic tract.

Visual changes are common after TBI of all severities, and include damage to multiple functions including oculomotor, visual acuity, eye-hand coordination, visual attention, and visual memory [32, 33]. In a subset of patients with traumatic vision loss after head trauma, the cause is injury to the optic nerve and its projections [34]. Damage specifically to the optic nerve is termed traumatic optic neuropathy (TON), and has been reported with all severities of TBI [35], including concussion [36]. TON can occur either by direct or indirect mechanisms, where direct injury is caused by a foreign body or broken bone directly damaging the optic nerve [35]. The etiology of indirect injury, on the other hand, is not well understood, but is thought to be due to transmission of traumatic forces through the bones of the skull into the optic nerve [37]. Consistent with this view, frontal loading of the human skull leads to deformation of the superior orbit and orbital canal [38], and the most common site of injury posterior to the globe in indirect injury is at the optic canal [39].

Several models of TON are in use, including optic nerve transection and optic nerve crush models [40]. These models are much more similar to direct TON than indirect [41]; however, indirect TON is likely more common clinically [35]. One model of indirect injury has recently been developed, using ultrasound to cause a focal injury at the optic canal [41], but it does not include traumatic forces and so may not replicate other important effects of a traumatic injury. Given these limitations, the model used in this study shows promise as a model of indirect TON, with injury likely occurring at the optic canal. In addition, there appears to be a neuroinflammatory response in our model, with activation of microglia in the optic tract, such as is seen in optic nerve crush injury models [42].

Microglia are the resident immune cells in the central nervous system, and are found throughout the brain [43]. They display a distinctive morphologic change in response to tissue injury [44], which occurs rapidly [45], and is thought to be a sign of neuroinflammation [46]. Iba-1 staining showed evidence of morphologic changes in microglia in the optic tracts, but not in other brain regions. This finding of morphologic changes in the optic tract, with apparent loss of ramifications and amoeboid morphology of the cell body, is consistent with neuroinflammation in the optic tracts. Interestingly, the inflammatory response does not appear to extend significantly to brain regions directly innervated by these axons. It is also interesting to note that the first subtle evidence of microglial activation emerges within 24 hours after injury, which precedes detectable change in either degeneration stain (Fluoro Jade B) or gliosis (GFAP staining). This finding may suggest that microglial activation precedes glial activation, a conclusion consistent with reports that retinal gliosis is mediated (and thus preceded) by microglial activation [47]. This is also consistent with previous findings in this model that blocking neuroinflammation prevents motor deficits induced by injury [6]. This view, however, does not explain the presence of astrocytosis in LGN and superior colliculus in the absence of microglial activation in these areas, which may thus occur via a different mechanism. Future studies will be needed to more fully understand the role of neuroinflammation in pathology of optic nerve injury in this model.

The histologic results in the current study are similar to findings in a mouse midline fluid percussion injury model of TBI, in which damage to the optic nerve is accompanied by microglial infiltration of the optic nerve [48, 49]. In the fluid percussion model, there is decreased innervation of the LGN at 4 days after injury, which is at least partially recovered by 10 to 20 days after injury [50]. Other groups have reported injury in the optic system after experimental blast TBI. In studies where optic nerve and central measures of damage were both performed, extensive damage was reported in multiple other brain areas [51–54]; thus the injury was not specific to the visual system. Visual system damage has also been reported using closed head injury models, mostly in repeat injury. In these studies, many reported injury in multiple non-optic areas [55–60]. A few studies also reported results in a single impact group, but either reported no histologic changes [58, 61], or did not examine thalamic areas or the SCN [59]. Several other groups have reported optic nerve damage, but did not examine central projections of the optic nerve for evidence of degeneration [62–64]. In sum, although this is not the first report of damage to the optic nerve/optic tract after TBI, it is, to our knowledge, the only single-injury TBI model to report results exclusively in the optic tract and its projections. Further, as noted above, our model appears to replicate at least partially the suspected mechanism for indirect TON in humans.

## Conclusion

We present a murine model of TBI that creates a reproducible traumatic injury to the optic nerve/optic tract. This injury is associated with microglial activation and astrocytosis, which affect regions directly innervated by the optic tract. It is simple to perform and reproducible, and appears to injure only a subset of optic tract neurons. Further, it appears to plausibly reproduce key features of traumatic optic neuropathy in humans. Thus, this model presents an opportunity to investigate the pathophysiology and treatment of traumatic axonal injury, particularly in the optic nerve.
